# Pediatric Maxillofacial Fractures: Patterns of Injury, Surgical Indications, and Treatment Outcomes: A Five-Year Retrospective Study

**DOI:** 10.3390/jcm15010019

**Published:** 2025-12-19

**Authors:** Krzysztof Gąsiorowski, Weronika Michalik, Jakub Bargiel, Tomasz Marecik, Julia Miaśkiewicz, Miłosz Saryusz-Romiszewski, Grażyna Wyszyńska-Pawelec, Michał Gontarz

**Affiliations:** 1 Department of Cranio-Maxillofacial Surgery, University Hospital, Jagiellonian University Medical College, Jagiellonian University, 30-688 Cracow, Poland; jakub.bargiel@uj.edu.pl (J.B.); tomasz.marecik@uj.edu.pl (T.M.); grazyna.wyszynska-pawelec@uj.edu.pl (G.W.-P.); michal.gontarz@uj.edu.pl (M.G.); 2 Students’ Scientific Group, Department of Cranio-Maxillofacial Surgery, University Hospital, Jagiellonian University Medical College, Jagiellonian University, 30-688 Cracow, Poland; weronika.michalik@student.uj.edu.pl (W.M.); julia.miaskiewicz@student.uj.edu.pl (J.M.); 3 Department of Pediatric Surgery, Institute of Pediatrics, Jagiellonian University Medical College, Jagiellonian University, 30-663 Cracow, Poland; milosz.saryusz-romiszewski@uj.edu.pl

**Keywords:** pediatric craniofacial trauma, facial fractures, epidemiology, maxillofacial surgery

## Abstract

**Background:** Pediatric craniofacial fractures represent a distinct clinical entity characterized by unique anatomical and developmental factors that differentiate them from adult facial trauma. Despite their relative rarity, these injuries pose diagnostic and therapeutic challenges due to the presence of active growth centers and the potential for long-term functional and esthetic sequelae. **Methods**: A retrospective observational study was conducted among pediatric patients aged 0–17 years treated for craniofacial fractures between 2020 and 2024 at the Department of Cranio-Maxillofacial Surgery, University Hospital in Kraków, Poland. Demographic data, injury mechanisms, fracture distribution, treatment modality, and associated injuries were analyzed. Multivariate logistic regression was applied to identify predictors of surgical intervention. **Results:** Ninety-eight patients met the inclusion criteria. The mean age was 12 years, with a male predominance. Midfacial fractures were most common, with orbital floor fractures representing the single most frequent injury. Surgical management was performed in 72 cases, predominantly using the transconjunctival approach and autologous bone grafting. Orbital floor fractures were identified as the only independent predictor of operative treatment (*p* < 0.05). Central nervous system trauma was the most frequent concomitant injury. No significant changes in etiology or fracture distribution were observed during the COVID-19 pandemic. **Conclusions:** Pediatric craniofacial trauma follows a reproducible, age- and mechanism-dependent pattern. Effective management requires individualized, growth-preserving, and function-oriented treatment strategies. Standardization of care protocols and multicenter prospective studies are essential to optimize outcomes and develop evidence-based, age-specific guidelines for the management and prevention of pediatric facial fractures.

## 1. Introduction

Pediatric craniofacial fractures represent a distinct clinical entity that differs substantially from adult facial trauma with respect to epidemiology, biomechanics, diagnostic challenges, and therapeutic principles. Although relatively uncommon—accounting for approximately 1% to 15% of all facial fractures—these injuries demand particular clinical attention due to their potential to result in long-term functional impairment, facial asymmetry, growth disturbances, and psychosocial sequelae if inadequately treated [1].

The lower incidence of facial fractures in children compared with adults can be attributed to unique anatomical and physiological characteristics, including a larger cranium-to-face ratio, greater elasticity of the facial skeleton, and thicker adipose and soft tissue coverage. Collectively, these features provide a degree of natural protection against fracture propagation [2]. Nevertheless, when fractures do occur, their management is often more complex than in adults, primarily because of the presence of active craniofacial growth centers, developing dentition, and varying bone remodeling capacities [3]. Pediatric facial bones contain a higher proportion of cancellous bone and demonstrate greater osteogenic potential, facilitating rapid healing but also predisposing to growth abnormalities if treatment is poorly timed or improperly executed [4].

Consequently, treatment protocols developed for adults cannot be directly applied to pediatric patients. Clinical decision-making must carefully balance the goals of anatomical reduction and stable fixation with the preservation of facial growth potential and normal functional development [5].

The etiology of pediatric facial fractures is strongly age-dependent. In younger children, falls and domestic accidents predominate, whereas in school-aged children and adolescents, road traffic collisions and sports-related injuries are more frequent [6]. Importantly, pediatric facial fractures are often associated with concomitant injuries, particularly to the central nervous system, cervical spine, and thoracoabdominal region. This underscores the importance of comprehensive trauma evaluation and multidisciplinary management involving pediatric surgeons, maxillofacial specialists, and neurosurgeons [7].

Despite increasing interest in pediatric craniofacial trauma, the current body of evidence on the epidemiology, management, and long-term outcomes of pediatric facial fractures remains heterogeneous. Substantial variability in diagnostic and therapeutic approaches persists across institutions and geographic regions [8]. Moreover, data from Central and Eastern Europe are particularly scarce, with limited high-quality studies assessing epidemiological trends and long-term functional or esthetic outcomes. This paucity of standardized evidence highlights the need for further multicenter, population-based research to optimize management strategies and establish evidence-based treatment guidelines.

## 2. Materials and Methods

This retrospective observational study included pediatric patients aged 0–17 years who sustained craniofacial fractures and received treatment between January 2020 and December 2024 at the Department of Cranio-Maxillofacial Surgery, University Hospital in Kraków, Poland. The study followed the principles of the Declaration of Helsinki.

A comprehensive review of electronic medical records was conducted to extract demographic variables (age and sex), mechanism of injury, anatomical location of the fracture, treatment modality (conservative vs. surgical), postoperative complications, and the presence of concomitant injuries. Only patients with complete clinical and radiological documentation were included. Patients presenting with isolated nasal bone fractures—typically managed by otolaryngologists in the Emergency Department—were excluded. Isolated soft-tissue injuries without skeletal involvement were also excluded.

Qualitative variables were presented as counts and percentages. Associations between categorical variables were assessed using the chi-square test with Yates’ continuity correction for 2 × 2 contingency tables. Fisher’s exact test was applied when expected cell counts were below the recommended threshold.

A multivariate logistic regression model was constructed to identify independent predictors associated with the need for surgical intervention. Variables entered into the model were selected based on a two-step approach: (1) univariate analysis, in which variables with *p* < 0.05 were considered for inclusion, and (2) clinical relevance, defined a priori based on established risk factors reported in pediatric craniofacial trauma literature. The final model included age group, mechanism of injury (high-energy vs. low-energy), presence of concomitant injuries, and fracture location categories. Odds ratios (ORs) with 95% confidence intervals (CIs) were calculated.

Because several fracture subtypes and mechanisms of injury represented small subgroups within the cohort, some regression estimates yielded wide confidence intervals. This limitation reflects the sample size and heterogeneity of fracture patterns in the pediatric population. As a result, the precision of certain effect estimates may be reduced, and these results should be interpreted with appropriate caution.

All statistical analyses were performed using R statistical software (version 4.5.0; R Foundation for Statistical Computing, Vienna, Austria).

## 3. Results

A total of 98 pediatric patients with facial fractures met the eligibility criteria for inclusion in this study. The mean patient age was 12 years, with adolescents aged 13–17 years representing the largest subgroup, followed by children aged 7–12 years and those younger than 6 years. This age-related distribution reflects a progressive increase in trauma exposure with advancing age. A clear male predominance was observed. The demographic and clinical characteristics of the study cohort are summarized in Table 1.

**Figure 1:**
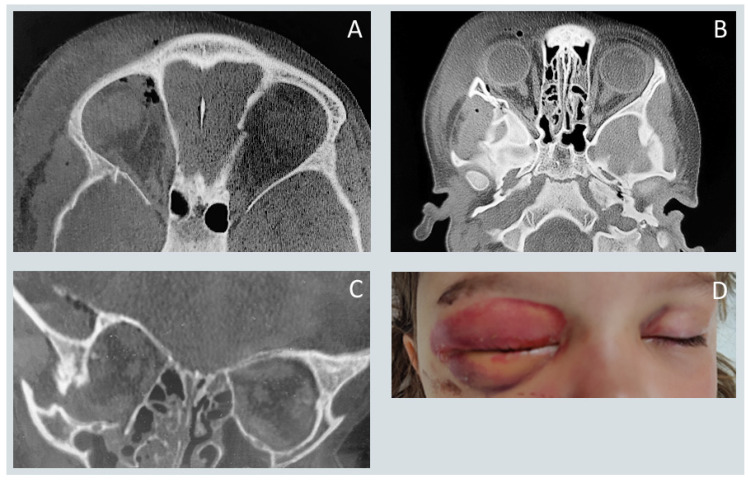
CT imaging (axial planes (**A**,**B**), and coronal plane (**C**)) reveals displacement of the zygomaticomaxillary complex consistent with a high-energy impact injury. Image (**D**) shows a 3.5-year-old child with marked periorbital edema and ecchymosis following a road traffic accident.

**Figure 2:**
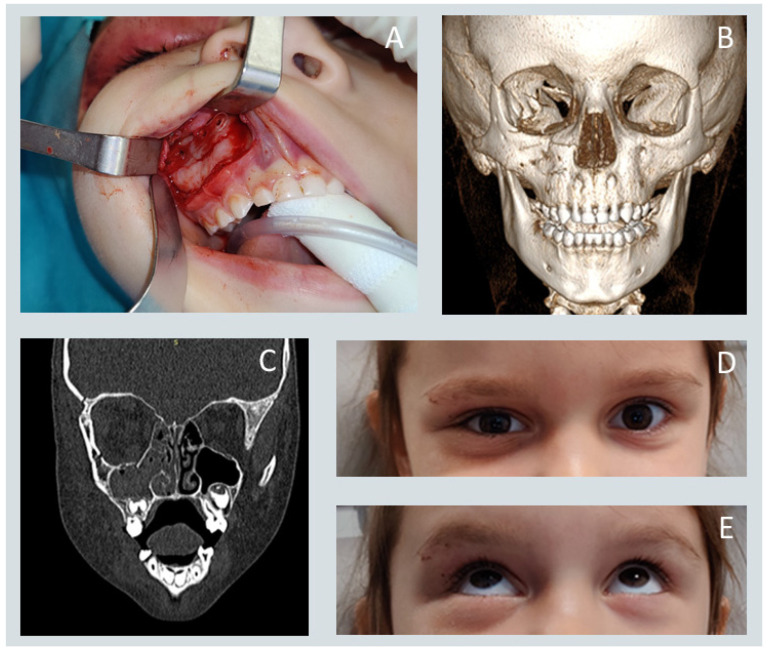
(**A**) Intraoperative photograph showing the reduced zygomaticomaxillary complex fracture with a resorbable plate in place. (**B**) Three-dimensional reconstruction from computed tomography. (**C**) Coronal-plane CT demonstrating proper alignment of the orbital floor. The displaced bony fragments were anatomically repositioned during perconjunctival approach. No bone graft was needed to reconstruct orbital floor (**D**,**E**) Clinical photographs illustrating normal extraocular motility.

Midfacial fractures constituted the majority of injuries, followed by fractures involving the lower and upper facial thirds. Figure 1 and Figure 2 present the surgical treatment of a patient with a zygomatic complex fracture. Among specific anatomical sites, orbital floor fractures were the most frequently diagnosed, exceeding mandibular and maxillary fractures as well as injuries to the mandibular condyle and zygomatic. Fractures of the frontal sinus, superior orbital rim or roof, and naso-orbito-ethmoidal (NOE) complex were rare.

Surgical management of orbital floor fractures was most commonly performed using the transconjunctival approach, which provides direct access to the orbital floor while avoiding external scarring and minimizing the risk of postoperative eyelid malposition. In cases with osseous defects, anatomical reconstruction was typically achieved using autologous bone grafts harvested from the anterior wall of the maxillary sinus. In younger children, however, the presence of developing permanent tooth buds contraindicated this donor site, necessitating alternative techniques such as repositioning displaced bone fragments or utilizing autologous calvarial grafts harvested from the parietal region.

Mandibular and maxillary fractures also represented a substantial proportion of injuries. Road traffic accidents were identified as the predominant etiological factor, followed by low-energy falls, reflecting the coexistence of both high- and low-energy trauma mechanisms in this pediatric population. Treatment strategies were individualized according to fracture morphology, degree of displacement, occlusal disturbance, and the presence of concomitant facial or systemic injuries. Conservative management (maxillomandibular fixation, soft diet) was preferred for nondisplaced fractures with preserved occlusion, whereas open reduction and internal fixation (ORIF) were indicated for displaced or unstable fractures.

**Figure 3:**
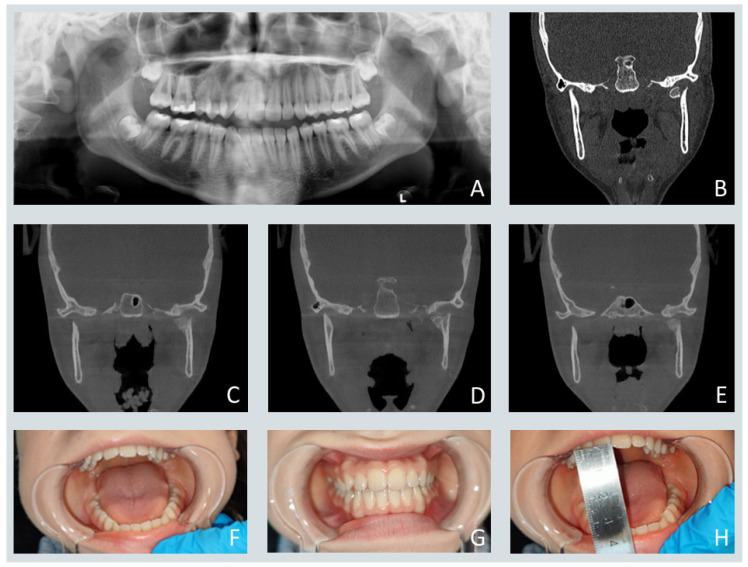
(**A**,**B**) Panoramic radiograph and coronal-plane CT scan demonstrating a mandibular head fracture located medial to the pole zone, without fragmentation and with partial vertical apposition, consistent with the 2014 Neff Classification. The letter L in image A indicates the patient’s left side. (**C**–**E**) Coronal-plane CT scans obtained three months post-injury showing progressive fracture healing. (**F**–**H**) Clinical photographs illustrating proper occlusion and normal mandibular movement following treatment.

Figure 3 illustrates a representative case of conservative treatment of a unilateral condylar head fracture in a 12-year-old patient, demonstrating satisfactory functional recovery and restoration of occlusion at three months post-injury.

A total of 72 patients underwent open reduction and internal fixation (ORIF). Titanium osteosynthesis plates were used in nearly all operative cases due to their superior biomechanical stability, whereas resorbable systems were applied selectively. Miniplate and microplate removal was performed in 19 patients approximately three months postoperatively, primarily for growth-related considerations. No postoperative infections were observed. One transient neuropraxia of the buccal branch of the facial nerve occurred and resolved completely without residual deficit.

As shown in Table 2, a statistically significant association was identified between patient age and fracture distribution. Superior orbital rim and orbital roof fractures were observed predominantly in children under six years of age, likely reflecting the prominent craniofacial contour and unique biomechanical vulnerability of the pediatric upper face. A strong correlation was also noted between age and mechanism of injury: falls were most common in younger children, traffic-related injuries in school-aged patients, and sports-related trauma and interpersonal violence in adolescents. These findings indicate that mechanism-specific injury patterns evolve predictably with age, underscoring the importance of age-adapted trauma prevention strategies.

Multivariate logistic regression analysis demonstrated that fractures of the upper third of the face were associated with an 84.6% reduction in the likelihood of surgical intervention compared with other fracture sites. Conversely, orbital floor fractures emerged as the only independent predictor of operative management (*p* < 0.05), reflecting their high functional significance due to the risk of extraocular muscle entrapment, diplopia, and persistent orbital deformity (Table 3).

As shown in Table 3, the multivariate logistic regression model showed that only orbital floor fracture of the analyzed characteristics is a significant independent predictor of the likelihood of potential surgical treatment.

Concomitant injuries were common in the study cohort. Central nervous system trauma represented the most frequent associated injury, followed by soft-tissue injuries, extremity fractures, and abdominal trauma. The mechanism of injury showed a significant association with the severity and distribution of concomitant trauma: road traffic accidents were most frequently associated with multisystem injuries, whereas sports-related trauma was largely confined to the craniofacial region (Table 4). These findings emphasize the necessity for comprehensive trauma evaluation and coordinated multidisciplinary management in pediatric patients presenting with facial fractures.

Finally, time-trend analysis revealed that the COVID-19 pandemic (2020–2024) did not significantly alter the etiological distribution of pediatric craniofacial injuries. Despite social restrictions and changes in activity patterns during national lockdowns, the mechanisms of injury remained stable, suggesting that pediatric facial trauma may be less sensitive to short-term public health measures than adult trauma profiles.

## 4. Discussion

Pediatric maxillofacial trauma constitutes a complex and clinically significant domain within craniofacial surgery, primarily due to the distinctive anatomical, developmental, and biomechanical characteristics of the growing facial skeleton. The present study reinforces the well-documented epidemiological pattern observed across multiple geographic regions: the incidence and severity of craniofacial fractures increase progressively with age and peak during adolescence. This gradient has been consistently reported in national and multicenter cohorts, including the large-scale database analyses by Allareddy et al. [9] and Imahara et al. [2], as well as more recent international studies [1,10,11,12,13]. Consistent with these findings, adolescents represented the largest subgroup in our cohort, followed by school-aged children and preschoolers—likely reflecting increasing independence, exposure to high-energy environments, and participation in organized sports with advancing age.

A pronounced male predominance was also observed, in line with the broader pediatric trauma literature [1,2,9,10,11,12]. The consistent replication of this pattern across diverse healthcare settings suggests that sex-related differences arise predominantly from behavioral rather than biological factors—namely, greater participation in contact sports, higher levels of unsupervised activity, and increased risk-taking behaviors among boys.

The mechanisms of injury exhibited a clear age-dependent transition. Falls from standing height predominated among preschoolers, while road-traffic incidents were most frequent in school-aged children, and sports-related trauma together with interpersonal violence were most common in adolescents. Similar age–mechanism correlations have been documented in large multicenter datasets [10,11] and in focused studies on pediatric sports injuries [8] and mandibular fractures [14]. These findings collectively support an age-stratified approach to injury prevention, including home safety and fall mitigation for young children, road-safety education for school-aged children, and enforcement of protective sports equipment and violence-prevention initiatives for adolescents.

With respect to anatomical distribution, midfacial fractures represented the majority of injuries in this series, consistent with previous multicenter observations indicating the susceptibility of the pediatric midface due to its retrusion, higher cranium-to-face ratio, and incomplete sinus pneumatization during early development [10,11]. We also identified a distinct age-specific pattern, with superior orbital rim and roof fractures occurring predominantly in children younger than six years. This observation aligns with developmental craniofacial anatomy described by Dryden et al. [15] and imaging-based studies by Hopper et al. [16], which emphasize the prominence of the frontal bone and delayed pneumatization of the frontal sinus in early childhood.

A notable finding of our study was the high proportion of orbital floor fractures, which emerged as the most common single fracture type. While earlier literature emphasized the mandible as the most frequently fractured bone in pediatric populations [2,9], recent studies demonstrate a growing burden of midfacial and orbital injuries [10,17]. As outlined by Coviello et al. [17], the pediatric orbital floor exhibits elastic behavior and may fail even under low-energy impacts, producing “trapdoor” fractures with extraocular muscle entrapment. Grant et al. [18] further demonstrated that even small, hinged floor defects may cause clinically significant ocular motility restriction or oculocardiac reflex, often with subtle radiological findings. Our results therefore support the contemporary trend toward an increased incidence of orbital fractures in pediatric trauma, potentially reflecting the influence of organized sports and blunt midfacial impacts.

In our cohort, orbital floor reconstruction was most frequently performed using the transconjunctival approach, which provides excellent exposure while minimizing visible scarring and postoperative eyelid malposition [19,20]. When reconstruction was indicated due to osseous defects, autologous bone grafts—most commonly harvested from the anterior wall of the maxillary sinus—were preferred, consistent with principles of biologically compatible, growth-preserving reconstruction [17,19,20]. In younger patients, this donor site was avoided to protect developing permanent tooth buds, as recommended by Grant et al. [18] and Harrison et al. [21]. In these cases, floor continuity was restored by repositioning native bone fragments or using parietal calvarial bone grafts, approaches aligned with established pediatric craniofacial reconstructive principles [17,18,20,21,22]. These techniques illustrate the importance of age-appropriate donor-site selection and meticulous surgical planning to safeguard future growth.

Fractures of the mandible and maxilla, including the condylar process, also represented a significant subset of injuries. Recent reports have underscored the risk of diagnostic errors leading to delayed management of mandibular fractures, highlighting the relevance of imaging indicators such as the “air sign” on CT or CBCT for detecting occult concomitant body fractures [23]. Treatment in our series emphasized restoration of function—occlusion, mandibular mobility, and stability—over purely anatomical correction, in accordance with pediatric management guidelines [14]. Titanium osteosynthesis systems were most frequently used, with resorbable materials applied selectively. This approach reflects current evidence demonstrating comparable safety profiles across fixation systems but a continued preference for titanium in load-bearing regions due to superior mechanical reliability [24]. Elective plate removal was performed in a subset of cases following early bone consolidation. Postoperative morbidity was minimal, limited to a single transient neuropraxia of the buccal branch of the facial nerve and no infectious complications—results consistent with contemporary pediatric series emphasizing careful soft-tissue handling and appropriately scaled fixation [20,24].

Multivariable analysis provided additional insight into clinical decision-making. Upper-third fractures were associated with a significantly lower likelihood of operative intervention, whereas orbital floor fractures were the only independent predictor of surgery. This is clinically logical, as upper-third fractures in children are typically nondisplaced and amenable to conservative management, while orbital floor injuries often necessitate surgical exploration due to the risk of diplopia, extraocular muscle entrapment, or oculocardiac reflex—widely accepted operative indications in pediatric orbital trauma [16,17,18]. These results support a functional and mechanism-driven triage strategy emphasizing ocular motility, occlusion, displacement, and growth potential over anatomical classification alone.

Concomitant injuries in our cohort reflected the energy spectrum of the causative mechanism. Patients involved in road-traffic accidents exhibited the highest rates of associated injuries, including limb and abdominal trauma, consistent with national database analyses linking high-energy mechanisms with multisystem involvement [2]. By contrast, sports-related injuries were largely confined to the craniofacial region, as expected for localized, moderate-force impacts [8,17]. Central nervous system trauma was the most frequent associated injury overall, consistent with literature emphasizing the close association between midfacial or orbital fractures and traumatic brain injury, warranting a low threshold for neuroimaging in such cases [25]. Moreover, recent studies confirm that the Facial Injury Severity Scale (FISS) remains a valuable tool for stratifying maxillofacial trauma severity and predicting surgical complexity and hospitalization duration, particularly in panfacial fractures [26].

The analysis of trauma patterns during the COVID-19 pandemic revealed no significant change in the etiology of pediatric craniofacial injuries between 2020 and 2024. While several studies in mixed-age populations reported altered trauma profiles during lockdowns [27], pediatric-focused investigations have shown relatively stable injury mechanisms [28]. These discrepancies likely reflect regional differences in restrictions, baseline activity patterns, and healthcare accessibility. Our findings suggest that pediatric facial trauma mechanisms are less sensitive to short-term societal disruptions than adult trauma profiles.

Overall, the present study integrates coherently with existing literature while contributing novel, region-specific data from Central and Eastern Europe. The observed trends-age gradient, male predominance, mechanism stratification, and increasing midfacial burden are consistent with international registry findings [1,2,9,10,11,12,13,14]. The operative approaches described, emphasizing transconjunctival access and autologous reconstruction tailored to age-specific anatomy, mirror best practices in pediatric craniofacial surgery [17,18,19,20,21,29]. Importantly, the identification of orbital floor fractures as independent predictors of surgical intervention underscores the need to prioritize functional outcomes and entrapment risk over anatomical site alone [30]. Collectively, these findings highlight the importance of mechanism-aware triage, age-adapted prevention strategies, and multidisciplinary care pathways to optimize outcomes and preserve facial growth potential in pediatric trauma patients.

## 5. Conclusions

Pediatric craniofacial trauma follows a consistent epidemiological pattern influenced by age, sex, and injury mechanism [5,31]. Although certain management principles mirror those used in adults, pediatric patients require a distinct, growth-conscious approach—the maxim “a child is not a small adult” remains fundamental. The anatomical plasticity of the developing facial skeleton and the presence of active growth centers necessitate individualized, function-oriented treatment strategies.

Optimal outcomes depend on accurate diagnosis, mechanism-aware planning, and prudent selection between conservative and surgical management to prevent long-term sequelae such as facial asymmetry, malocclusion, or diplopia. The observed correlation between trauma mechanism and associated injuries underscores the importance of comprehensive trauma evaluation and multidisciplinary care. However the single-center setting may limit generalizability

Future progress relies on standardized treatment algorithms, refined diagnostic tools, and multicenter prospective studies to develop evidence-based, age-specific management and prevention strategies that preserve both functional and developmental integrity.

## Figures and Tables

**Table 1:** Baseline demographic and clinical features of pediatric patients with craniofacial fractures.

Parameter		Total (N = 98)
Age [years]	Mean (SD)	12.68 (4.36)
	Median (quartiles)	14 (11–16)
	Range	1–18
	n	98
Age	0–6	11 (11.22%)
	7–12	27 (27.55%)
	13–18	60 (61.22%)
Gender	Male	76 (77.55%)
	Female	22 (22.45%)
Fracture location	Fracture in the lower face	35 (35.71%)
	Fracture in the middle third of the face	63 (64.29%)
	Fracture in the upper face	13 (13.27%)
Detailed location of the fracture	Mandibular fracture (corpus and angle)	24 (24.49%)
	Fracture of the mandibular condyle	16 (16.33%)
	Maxillary fracture	23 (23.47%)
	Fracture of the zygomatic bone	16 (16.33%)
	Orbital floor fracture	33 (33.67%)
	NOE fracture	1 (1.02%)
	Nasal fracture	19 (19.39%)
	Sinus/frontal bone fracture	10 (10.20%)
	Fracture of the roof/upper rim of the orbit	6 (6.12%)
Mechanism of injury	Fall	24 (24.49%)
	Traffic accident	37 (37.76%)
	Sports injury	16 (16.33%)
	Assault	18 (18.37%)
	Other	3 (3.06%)
Treatment	Surgical	75 (76.53%)
	Conservative	23 (23.47%)
Accompanying injuries	Skin injuries	30 (30.61%)
	CNS injuries	32 (32.65%)
	Abdominal injuries	7 (7.14%)
	Limb injuries	12 (12.24%)
	Spinal injuries	4 (4.08%)
	No accompanying injuries	41 (41.84%)

**Table 2:** Correlation between patient age and specific fracture sites (The total number of fractures listed in the table exceeds the number of patients, as individual subjects could sustain multiple distinct fracture sites).

Detailed Location of the Fracture	Age			*p*
	0–6 Years (11 Patients)	7–12 Years (27 Patients)	13–18 Years (60 Patients)	
Mandibular fracture (corpus and angle)	3 (27.27%)	8 (29.63%)	13 (21.67%)	*p* = 0.689
Mandibular condyle fracture	1 (9.09%)	5 (18.52%)	10 (16.67%)	*p* = 0.923
Maxillary fracture	3 (27.27%)	5 (18.52%)	15 (25.00%)	*p* = 0.774
Zygomatic bone fracture	0 (0.00%)	5 (18.52%)	11 (18.33%)	*p* = 0.394
Orbital floor fracture	4 (36.36%)	9 (33.33%)	20 (33.33%)	*p* = 1
NOE fracture	0 (0.00%)	1 (3.70%)	0 (0.00%)	*p* = 0.388
Nose fracture	2 (18.18%)	4 (14.81%)	13 (21.67%)	*p* = 0.864
Sinus/frontal bone fracture	1 (9.09%)	3 (11.11%)	6 (10.00%)	*p* = 1
Fracture of the roof/upper rim of the orbit	3 (27.27%)	0 (0.00%)	3 (5.00%)	*p* = 0.017

**Table 3:** Multivariate logistic regression analysis identifying predictors of surgical management in pediatric patients with craniofacial fractures.

Feature		N	n	OR	95% CI		*p*
Age	0–6 years	11	8	1	ref.		
	7–12 years	27	20	0.475	0.06	3.754	0.48
	13–17 years	60	47	0.756	0.112	5.11	0.774
Mandibular fracture	No	74	55	1	ref.		
	Yes	24	20	2.041	0.463	8.992	0.346
Fracture of the mandibular condyle	No	82	64	1	ref.		
	Yes	16	11	0.628	0.151	2.617	0.523
Jaw fracture	No	75	58	1	ref.		
	Yes	23	17	0.797	0.217	2.934	0.733
Fracture of the zygomatic bone	No	82	62	1	ref.		
	Yes	16	13	1.844	0.337	10,092	0.48
Fracture of the orbital floor	No	65	46	1	ref.		
	Yes	33	29	3.686	0.926	14.67	0.05
Nose fracture	No	79	59	1	ref.		
	Yes	19	16	1.195	0.242	5.895	0.827
Fracture of the sinus/frontal bone	No	88	70	1	ref.		
	Yes	10	5	0.36	0.075	1.727	0.202
Fracture of the roof/upper rim of the orbit	No	92	73	1	ref.		
	Yes	6	2	0.119	0.014	1.01	0.051

**Table 4:** Association between mechanism of injury and type of associated injuries.

Accompanying Injuries	Mechanism of Injury					*p*
	Fall (N = 24)	Traffic Accident (N = 37)	Sports Injury (N = 16)	Assault (N = 18)	Other (N = 3)	
Skin injuries	9 (37.50%)	15 (40.54%)	2 (12.50%)	2 (11.11%)	2 (66.67%)	*p* = 0.034
CNS injuries	7 (29.17%)	15 (40.54%)	2 (12.50%)	8 (44.44%)	0 (0.00%)	*p* = 0.163
Abdominal injuries	0 (0.00%)	7 (18.92%)	0 (0.00%)	0 (0.00%)	0 (0.00%)	*p* = 0.03
Limb injuries	1 (4.17%)	11 (29.73%)	0 (0.00%)	0 (0.00%)	0 (0.00%)	*p* = 0.003
Spinal injuries	1 (4.17%)	3 (8.11%)	0 (0.00%)	0 (0.00%)	0 (0.00%)	*p* = 0.726
No accompanying injuries	10 (41.67%)	8 (21.62%)	12 (75.00%)	10 (55.56%)	1 (33.33%)	*p* = 0.003

## Data Availability

The original contributions presented in this study are included in the article. Further inquiries can be directed to the corresponding author.
